# Spatiotemporal multilevel joint modeling of longitudinal and survival outcomes in end-stage kidney disease

**DOI:** 10.1007/s10985-024-09635-w

**Published:** 2024-10-04

**Authors:** Esra Kürüm, Danh V. Nguyen, Qi Qian, Sudipto Banerjee, Connie M. Rhee, Damla Şentürk

**Affiliations:** 1 Department of Statistics, University of California, Riverside, CA 92521 USA; 2 Department of Medicine, University of California Irvine, Orange, CA 92868 USA; 3 Department of Biostatistics, University of California, Los Angeles, CA 90095 USA; 4 Department of Medicine, University of California, Los Angeles, CA 90095 USA; 5 Nephrology Section, VA Greater Los Angeles Health Care System, Los Angeles, CA 90073 USA

**Keywords:** Conditional autoregressive model, End-stage kidney disease, Markov chain Monte Carlo, United States renal data system, Varying-coefficient models, 62Hxx, 62Nxx, 62P10

## Abstract

Individuals with end-stage kidney disease (ESKD) on dialysis experience high mortality and excessive burden of hospitalizations over time relative to comparable Medicare patient cohorts without kidney failure. A key interest in this population is to understand the time-dynamic effects of multilevel risk factors that contribute to the correlated outcomes of longitudinal hospitalization and mortality. For this we utilize multilevel data from the United States Renal Data System (USRDS), a national database that includes nearly all patients with ESKD, where repeated measurements/hospitalizations over time are nested in patients and patients are nested within (health service) regions across the contiguous U.S. We develop a novel spatiotemporal multilevel joint model (STM-JM) that accounts for the aforementioned hierarchical structure of the data while considering the spatiotemporal variations in both outcomes across regions. The proposed STM-JM includes time-varying effects of multilevel (patient- and region-level) risk factors on hospitalization trajectories and mortality and incorporates spatial correlations across the spatial regions via a multivariate conditional autoregressive correlation structure. Efficient estimation and inference are performed via a Bayesian framework, where multilevel varying coefficient functions are targeted via thin-plate splines. The finite sample performance of the proposed method is assessed through simulation studies. An application of the proposed method to the USRDS data highlights significant time-varying effects of patient- and region-level risk factors on hospitalization and mortality and identifies specific time periods on dialysis and spatial locations across the U.S. with elevated hospitalization and mortality risks.

**Supplementary Information:**

The online version contains supplementary material available at 10.1007/s10985-024-09635-w.

## Introduction

The latest national data from the United States renal data system shows that about 130,500 ESKD patients transitioned to dialysis in 2020 in the U.S. and ESKD affected nearly 808,000 individuals as of 2020, with about 70% of patients on dialysis, a life-sustaining treatment (USRDS 2022). Also, although ESKD accounts for less than 2% of the Medicare beneficiaries, ESKD consumes about 7% ($$\sim$$ $50 billions in 2018) of total Medicare budget (USRDS 2022; National Kidney Foundation 2022). The mortality risk among individuals on dialysis is substantially higher than most other morbid populations, including Medicare populations with cancer, diabetes, or cardiovascular disease. Furthermore, due to the nature of dialysis treatment and the high burden of complex comorbid conditions, patients on dialysis experience frequent hospitalizations (about twice per year), a major source of morbidity and mortality (USRDS 2022). Therefore, hospitalization and mortality are intricately related outcomes that impact patients’ quality of life, remaining lifespan, and healthcare cost. Elucidating the time-dynamic effects of both patient- and region-level risk factors, as well as better understanding of the specific time periods (after patients transition to dialysis) of higher risk for adverse events may inform patient management and treatment strategies.

Towards this goal, we analyze data from the USRDS, a large national database containing information on potential patient risk factors (including baseline demographics and comorbidities), linked to region-level covariates (i.e., urbanicity, area deprivation index [ADI] and medical underservice index [MUI]). One challenge in the analysis of this data is due to its three-level hierarchical structure: longitudinal hospitalizations are nested within patients and patients are nested within regions across the U.S. Furthermore, previous analysis of the aggregate USRDS data demonstrated significant spatiotemporal variations in both hospitalization and mortality risks across the U.S. as well as over the course of dialysis treatment. For patients with ESRD, dialysis is a long-term life-sustaining treatment for the duration of their lives or until successful kidney transplantation. Since the patient’s clinical conditions and needs may change as they remain on dialysis, their hospitalization and mortality risks also vary temporally, post-dialysis transition. These temporal changes have been reported, including higher hospitalization and mortality rates within the first year and first six months on dialysis, respectively (Estes et al. 2018; Li et al. 2018), and after major medical events such as infections (Estes et al. 2014; Mohammed et al. 2012). Kürüm et al. (2022) examined time-varying effects of risk factors on both outcomes. Although these studies elucidated the temporal patterns in the data and demonstrated time-varying effects of risk factors, their methods were limited to studying temporal variations.

Previous works (Li et al. 2021, 2022; USRDS 2022; Qian et al. 2023, 2024) considered the spatial variations in ESKD hospitalization and mortality rates and identified spatial clusters of health services areas (HSAs: geographic regions with relatively self-contained infrastructure for the provision of hospital care) with high rates. However, these approaches considered the unit of analysis as the aggregated rate of hospitalization (or mortality) over a dialysis facility within a spatial region (HSA) and with covariates also aggregated per dialysis facility. Spatiotemporal joint modeling of these outcomes at the individual (patient) level while accounting for the spatiotemporal trends remains incomplete. Therefore, in this work, we develop a joint modeling framework for the correlated outcomes of longitudinal hospitalization and survival at the subject level, accounting for all features of the USRDS data (three-level hierarchy and spatiotemporal variations) simultaneously, to target more granular estimates of the time-varying effects of the multilevel risk factors. The multilevel joint model incorporates the multilevel risk factors more intrinsically than methods using aggregated data and provides a more targeted study of the reasons behind the spatiotemporal variations of hospitalization and mortality risks, which could also be partially due to patient- or region-level risk factors.

Joint modeling of longitudinal and survival outcomes has been studied extensively over the last several decades. Tsiatis and Davidian (2004) present a comprehensive review of joint modeling techniques prior to 2004, and Rizopoulos (2012) provides details on estimation and inference along with implementation in R. A more recent review published by Gould et al. (2015) describes the latest methodologies and discusses issues with implementation and interpretation. The most popular approach to jointly modeling longitudinal and survival outcomes is the shared-parameter framework, where the dependency between the outcomes is incorporated via shared subject-level random effects (Wulfsohn and Tsiatis 1997; Henderson et al. 2000; Tsiatis and Davidian 2001; Song et al. 2002; Hsieh et al. 2006; Rizopoulos et al. 2008; Mauff et al. 2020).

Although many approaches on joint modeling are proposed for a two-level hierarchy, that is, longitudinal measurements nested within subjects, only a few studies consider a three-level data structure (with longitudinal measurements nested in subjects and subjects nested in a higher clustering unit). Liu et al. (2008) and Brilleman et al. (2019) proposed multilevel joint models for a three-level hierarchy, yet their works are limited to modeling the effects of subject-level risk factors. The works by Kürüm et al. (2021, 2022) extended the joint modeling framework to include the multilevel risk factors, with the latter incorporating time-varying effects of these risk factors. However, none of these multilevel joint models accounted for the spatial correlations in the data. The few works (Martins et al. 2016, 2017) that address this concern only focus on the spatial dependency within the survival response, which is modeled via the intrinsic conditional autoregressive (ICAR) structure, and are limited to modeling the time-invariant effects in a two-level hierarchical structure (repeated measurements nested within subjects).

In this paper, to address the aforementioned scientific and methodology gaps in knowledge, we propose a novel spatiotemporal multilevel joint model (STM-JM). The main contribution of our joint modeling framework is incorporating all key features of a complex data set, namely, the multilevel structure and spatiotemporal variations, while including multilevel covariates to study time-varying effects of both subject- and region-level risk factors. Our approach relies on the shared-parameter framework such that the dependency between the longitudinal and survival outcomes is modeled via multilevel REs at both levels of the hierarchy (subject and region). We impose a multivariate conditional autoregressive (CAR) correlation structure on the region-level REs to capture the remaining spatial correlation in the longitudinal and survival outcomes over regions after accounting for time-varying effects of subject- and region-level risk factors and subject-level dependencies. Note that the assumed region-level spatial random effects are more general than the spatial random effects of Martins et al. (2016, 2017) for multiple reasons. First, spatial random effects are included in modeling both outcomes, not only the survival outcome. In addition, the multilevel CAR modeling proposed can accommodate more general spatial correlations than those that can be modeled by ICAR. CAR modeling includes a spatial smoothing tuning parameter that can be estimated from the data, whereas ICAR is a special case of CAR where this tuning parameter is fixed (Wall 2004). To study the time-varying effects of multilevel risk factors, we utilize varying-coefficient models (Cleveland et al. 1992; Hastie and Tibshirani 1993; Cai et al. 2000; Li et al. 2018), which are widely used to estimate time-varying effects in longitudinal studies. Estimation and inference are performed via a Bayesian framework based on Markov Chain Monte Carlo (MCMC), where the time-varying coefficients are estimated via thin-plate splines (Crainiceanu et al. 2005).

The remainder of this paper is organized as follows. The proposed STM-JM formulation, along with the Bayesian estimation and inference, are described in Sect. 2. Section 3 presents simulation studies demonstrating the finite sample behavior of our proposed estimation and inference. The method is illustrated with a joint modeling of longitudinal hospitalization and survival of patients with ESKD using the USRDS data in Sect. 4. We conclude with a brief discussion in Sect. 5. Finally, R codes, documentation, example data, and instructions for fitting the proposed STM-JM are made publicly available at https://github.com/esrakurum/STM-JM.

## Spatiotemporal multilevel joint models

### Model specification

Let $Y_{ij}(t)$ denote the longitudinal outcome for the *j*th subject (patient), $j=1, \ldots , n_i$, at the *i*th region (HSA), $i=1,\ldots ,n$, at time *t*, where $Y_{ij}(t)$ might not necessarily be continuous. In our application to the USRDS data, the time index *t* is taken to be time (days) starting from when a patient transition to dialysis. For the survival outcome, the true and observed event (death) times are denoted by $T^{*}_{ij}$ and $T_{ij}$, respectively, for subject *j* at region *i*. The observed event time is defined as the minimum of the potential censoring time $C_{ij}$ and $T_{ij}^{*}$. The event indicator is defined as $\delta _{ij}= I (T_{ij}^{*}\le C_{ij})$, where $I (\cdot )$ is the indicator function and $\delta _{ij}=0$ corresponds to right censoring. We assume that the censoring mechanism is noninformative; that is, when conditioned on the observed covariates, it is independent of the longitudinal process.

To study the time-varying effects of risk factors on each outcome (hospitalization and survival), the proposed longitudinal and survival submodels include time-varying coefficients associated with each predictor,

1 $$\begin{aligned} m_{ij}(t) = E\lbrace Y_{ij}(t) |{\textbf{X}}_{ij}, {\textbf{Z}}_{i}, u_{1ij}, \nu _{1i} \rbrace= & g^{-1}\lbrace {\textbf{X}}_{ij}^\textrm{T}\varvec{\beta }_y(t) + {\textbf{Z}}_{i}^\textrm{T}\varvec{\gamma }_y(t) + u_{1ij} + \nu _{1i}\rbrace \nonumber \\ h_{ij}(t \mid {\textbf{X}}_{ij}, {\textbf{Z}}_{i},u_{2ij}, \nu _{2i})= & h_0(t)\exp \lbrace {\textbf{X}}_{ij}^\textrm{T}\varvec{\beta }_s(t) + {\textbf{Z}}_{i}^\textrm{T}\varvec{\gamma }_s(t) + u_{2ij} + \nu _{2i}\rbrace , \end{aligned}$$

where $g(\cdot )$ denotes the link function, $h_0(t)$ is the baseline hazard, and ${\textbf{X}}_{ij} = (X_{ij1}, \ldots ,$ $X_{ijP})^\textrm{T}$ and ${\textbf{Z}}_{i}= (Z_{i1},\ldots , Z_{iQ})^\textrm{T}$ are the subject- and region-level predictors, respectively. (We note that the covariate vectors, ${\textbf{X}}_{ij}$ and ${\textbf{Z}}_i$, need not be the same in the longitudinal and survival submodels in applications generally, although for convenience we keep them the same in (1).) The corresponding time-varying coefficients are denoted as $\varvec{\beta }_* (t)=\lbrace \beta _{* 1}(t),\ldots , \beta _{* P}(t)\rbrace ^\textrm{T}$ and $\varvec{\gamma }_* (t)=\lbrace \gamma _{*1}(t),\ldots , \gamma _{*Q}(t)\rbrace ^\textrm{T}$, respectively, in each submodel (with $*$ denoting *y* or *s*). For our motivating data application, $Y_{ij}(t)$ is a binary longitudinal outcome defined as the indicator of at least one hospitalization in a 3-month follow-up window with midpoint *t* for the subject *j* at region *i* and thus, the link function takes the form of the logit link $g(p) = \log \lbrace p/(1-p) \rbrace$.

The association between the outcomes is established via the subject- and region-level random effects (REs). Let ${\textbf{u}}_{ij} = (u_{1ij}, u_{2ij})^\textrm{T}$ be the vector of subject-level REs that is assumed to follow a multivariate normal distribution such that ${\textbf{u}}_{ij} \sim N( {\textbf{0}}, \varvec{\varSigma }_u)$ with $$\varvec{\varSigma }_u = \left( \begin{array}{lr} \sigma _{u_1}^2 & \sigma _{u} \\ \sigma _{u} & \sigma _{u_2}^2 \end{array} \right)$$, $\sigma _u = \rho _u \sigma _{u_1}\sigma _{u_2}$, $\rho _u$ denoting the correlation coefficient. We define $\varvec{\nu }= (\varvec{\nu }_{1}^\textrm{T}, \varvec{\nu }_{2}^\textrm{T})^\textrm{T}$ with $\varvec{\nu }_{1} = (\nu _{11}, \ldots , \nu _{1n})^\textrm{T}$ and $\varvec{\nu }_{2} = (\nu _{21}, \ldots , \nu _{2n})^\textrm{T}$ as the region-level REs in the longitudinal and survival submodels, respectively. Note that we assume that subject- and region-level REs are independent.

To capture the spatial correlation while accounting for the dependencies between the longitudinal and survival outcomes, we impose a multivariate conditional autoregressive (MCAR) model among region-level REs. More specifically, let $\varvec{\varSigma }_b$ and $(\varvec{\varSigma }_{w})_\ell$ ($\ell =1,2$) denote between-outcome and within-outcome matrices, respectively, where $\varvec{\varSigma }_b$ is the non-spatial covariance capturing dependencies between the two outcomes, whereas $(\varvec{\varSigma }_{w})_\ell =({\textbf{D}}-\alpha _\ell {\textbf{W}})^{-1}$ is the spatial covariance representing the spatial correlations across regions within each outcome. Note that $\alpha _\ell$ is the spatial smoothing parameter that we allow to be different for each outcome. Furthermore, ${\textbf{D}}$ is an $n\times n$ diagonal matrix with diagonal elements $d_i$ denoting the total number of neighbors of the *i*th region, and ${\textbf{W}}=\{w_{ii^\prime }\}$ is the $n\times n$ adjacency matrix that describes the neighborhood structure of the regions such that $w_{ii} = 0$ by convention, $w_{i i^\prime } = 1$ if regions *i* and $i^\prime$ ($i \ne i^\prime$) are neighbors, and $w_{i i^\prime } = 0$ otherwise. Then an MCAR($\alpha _1, \alpha _2, \varvec{\varSigma }_b$) correlation structure (Carlin and Banerjee 2003; Gelfand and Vounatsou 2003) is induced among region-level REs $\varvec{\nu }$ as follows: $\varvec{\nu }\sim {\mathcal {N}}(0, \varvec{\varSigma }_{\varvec{\nu }})$, where

2 $$\begin{aligned} \varvec{\varSigma }_\nu = \text {Bdiag} \left\{ ({\widetilde{\varvec{\varSigma }}}_{w})_{1}, ({\widetilde{\varvec{\varSigma }}}_{w})_{2}\right\} \left( \varvec{\varSigma }_b \otimes {\textbf{I}}\right) \text {Bdiag} \left\{ ({\widetilde{\varvec{\varSigma }}}_{w})_{1}^\textrm{T}, ({\widetilde{\varvec{\varSigma }}}_{w})^\textrm{T}_{2}\right\} . \end{aligned}$$

In (2), $\text {Bdiag}(\cdot )$ denotes a block diagonal matrix, ${\textbf{I}}$ is an $n \times n$ identity matrix, and $({\widetilde{\varvec{\varSigma }}}_{w})_{\ell }$ is the lower triangular matrix of the within-outcome matrix $({\varvec{\varSigma }}_{w})_{\ell }=({\textbf{D}}-\alpha _\ell {\textbf{W}})^{-1}$ such that $({\varvec{\varSigma }}_{w})_{\ell } = ({\widetilde{\varvec{\varSigma }}}_{w})_{\ell } ({\widetilde{\varvec{\varSigma }}}_{w})_{\ell }^\textrm{T}$ via the Cholesky decomposition.

In terms of our data application, the MCAR covariance structure presented in (2) allows us to account for two sources of covariance in the USRDS data: between-outcome dependency and within-outcome dependency. Between-outcome dependency captures the non-spatial correlation between the two outcomes (hospitalization and survival) within a region. By incorporating this dependency into the covariance structure, we account for the potential influence of one outcome on the other within the same region. Within-outcome dependency addresses spatial correlations across regions within each outcome exhibited due to shared practice patterns/factors (that is, spatial autocorrelation). This decomposition into between- and within-outcome dependencies is a commonly used structure due to its ease of interpretation.

### Estimation and inference

We propose to estimate the parameters in our joint modeling framework via a Bayesian estimation procedure and derive posterior inferences via a Markov Chain Monte Carlo (MCMC) algorithm. To formulate the model estimation and inference let $\varvec{\theta }(t) = \lbrace \varvec{\beta }(t), \varvec{\gamma }(t), \varvec{\theta }_{RE}, \varvec{\theta }_{h0}\rbrace ^\textrm{T}$ be the full parameter vector, where $\varvec{\beta }(t) = \lbrace \varvec{\beta }_y^\textrm{T}(t), \varvec{\beta }_s^\textrm{T}(t)\rbrace$ with $\varvec{\beta }_* (t)=\lbrace \beta _{* 1}(t),\ldots , \beta _{* P}(t)\rbrace ^\textrm{T}$ and $\varvec{\gamma }(t) = \lbrace \varvec{\gamma }_y^\textrm{T}(t), \varvec{\gamma }_s ^\textrm{T}(t) \rbrace$ with $\varvec{\gamma }_* (t)=\lbrace \gamma _{*1}(t),\ldots ,$ $\gamma _{*Q}(t)\rbrace ^\textrm{T}$ are the time-varying coefficients in both submodels ($*$ denoting *y* or *s*). Also, let $\varvec{\theta }_{RE} = (\sigma ^2_{u_1}, \sigma ^2_{u_2}, \rho _u, \alpha _1, \alpha _2, \varvec{\theta }_{\varSigma _b})$ be the parameters corresponding to subject- and region-level REs with $\varvec{\theta }_{\varSigma _b}$ denoting parameters associated with the between-outcome covariance matrix $\varvec{\varSigma }_b$ (2), and $\varvec{\theta }_{h_0}$ contains parameters used in modeling the baseline hazard function. We employ thin-plate splines in estimation of the baseline hazard function as well as the varying coefficient functions as outlined below.

The joint likelihood is derived under the conditional independence assumption, that is, the subject- and region-level REs account for the association between the two outcomes, and given the REs, the outcomes are independent. Furthermore, we assume that, in addition to the time-varying effects, the REs also contribute to modeling the correlation between the longitudinal measurements within a subject, leading to the conditional likelihood

$$\begin{aligned} p\lbrace {\textbf{Y}}_{ij}, T_{ij}, \delta _{ij} \mid {\textbf{u}}_{ij}, \varvec{\nu }_i;\varvec{\theta }(t)\rbrace= & p\lbrace {\textbf{Y}}_{ij} \mid u_{1ij}, \nu _{1i}; \varvec{\theta }(t)\rbrace \,\, p\lbrace T_{ij}, \delta _{ij} \mid u_{2ij}, \nu _{2i}; \varvec{\theta }(t)\rbrace \nonumber \\ p\lbrace {\textbf{Y}}_{ij} \mid u_{1ij}, \nu _{1i}; \varvec{\theta }(t) \rbrace= & \prod _{k=1}^{n_{ij}} p \lbrace Y_{ijk} \mid u_{1ij}, \nu _{1i};\varvec{\theta }(t) \rbrace , \end{aligned}$$

where for the *i*th region, $\varvec{\nu }_i = (\nu _{1i}, \nu _{2i})^\textrm{T}$ is the vector of region-level REs, ${\textbf{u}}_{ij} = (u_{1ij}, u_{2ij})^\textrm{T}$ is the vector of subject-level REs for the *j*th patient, and ${\textbf{Y}}_{ij}=(Y_{ij1}, \ldots ,$ $Y_{ijn_{ij}})^{\textrm{T}}$ denotes the $n_{ij}\times 1$ vector of longitudinal outcomes for the *j*th patient with $Y_{ijk} = Y_{ij}(t_{ijk})$, $k=1, \ldots , n_{ij}$ and $t_{ijk}$ denoting the midpoint of the *k*th three-month interval in the follow-up period. Therefore, the posterior distribution is derived as

3 $$\begin{aligned} p \lbrace \varvec{\theta }(t), {\textbf{u}}_{ij}, \varvec{\nu }_i \mid {\textbf{Y}}_{ij}, T_{ij}, \delta _{ij}\rbrace\propto & p\lbrace {\textbf{Y}}_{ij}, T_{ij}, \delta _{ij} \mid {\textbf{u}}_{ij}, \varvec{\nu }_i, \varvec{\theta }(t)\rbrace p \lbrace {\textbf{u}}_{ij}, \varvec{\nu }_i, \varvec{\theta }(t) \rbrace \nonumber \\ \propto & \prod _{k=1}^{n_{ij}} p\lbrace Y_{ijk}\mid u_{1ij}, \nu _{1i}, \varvec{\theta }(t) \rbrace p\lbrace T_{ij}, \delta _{ij} \mid u_{2ij}, \nu _{2i}, \varvec{\theta }(t) \rbrace \nonumber \\ & \times \, p\lbrace {\textbf{u}}_{ij}, \varvec{\nu }_{i}\mid \varvec{\theta }(t) \rbrace p\lbrace \varvec{\theta }(t) \rbrace . \end{aligned}$$

In our data application, where the longitudinal outcome is binary, the likelihood contribution from the longitudinal submodel takes the form

4 $$\begin{aligned} p\lbrace {\textbf{Y}}_{ij} \mid u_{1ij}, \nu _{1i}, \varvec{\theta }(t) \rbrace = \prod _{k=1}^{n_{ij}} \dfrac{\exp \left[ \lbrace {\textbf{X}}_{ij}^\textrm{T}\varvec{\beta }_y(t_{ijk}) + {\textbf{Z}}_{i}^\textrm{T}\varvec{\gamma }_y(t_{ijk})+u_{1ij} +\nu _{1i} \rbrace Y_{ijk} \right] }{ 1+ \exp \lbrace {\textbf{X}}_{ij}^\textrm{T}\varvec{\beta }_y(t_{ijk}) +{\textbf{Z}}_{i}^\textrm{T}\varvec{\gamma }_y(t_{ijk}) +u_{1ij} +\nu _{1i} \rbrace }. \end{aligned}$$

Moreover, the likelihood contribution of the survival submodel is

5 $$\begin{aligned} p\lbrace T_{ij}, \delta _{ij} \mid u_{2ij}, \nu _{2i}, \varvec{\theta }(t)\rbrace= & \left[ h_0(T_{ij}) \exp \lbrace {\textbf{X}}_{ij}^\textrm{T}\varvec{\beta }_s(T_{ij}) + {\textbf{Z}}_{i}^\textrm{T}\varvec{\gamma }_s(T_{ij}) + u_{2ij} +\nu _{2i} \rbrace \right] ^{\delta _{ij}}\nonumber \\ & \times \exp \left[ -\int _{0}^{T_{ij}} h_0(v) \exp \lbrace {\textbf{X}}_{ij}^\textrm{T}\varvec{\beta }_s(v) + {\textbf{Z}}_{i} ^\textrm{T}\varvec{\gamma }_s(v) +u_{2ij} +\nu _{2i} \rbrace dv \right] . \end{aligned}$$

Since the integral in (5) does not have a closed-form solution, a numerical approximation is employed. Common choices are Simpson’s and Gaussian quadrature rules; here we use the latter, in particular, a 15-point Gauss-Kronrod rule (Kahaner et al. 1989).

For the subject and region-level REs, under the assumption that they are uncorrelated, we write their joint density in (3) as $p\lbrace {\textbf{u}}_{ij}, \varvec{\nu }_{i}\mid \varvec{\theta }(t) \rbrace = p\lbrace {\textbf{u}}_{ij} \mid \varvec{\theta }(t) \rbrace p\lbrace \varvec{\nu }_{i} \mid \varvec{\theta }(t) \rbrace$. As mentioned in Sect. 2.1, we assume both REs follow a normal distribution ${\textbf{u}}_{ij} \sim N( {\textbf{0}}, \varvec{\varSigma }_u)$ with $$\varvec{\varSigma }_u = \left( \begin{array}{lr} \sigma _{u_1}^2 & \sigma _{u} \\ \sigma _{u} & \sigma _{u_2}^2 \end{array} \right)$$ and $\sigma _u = \rho _u \sigma _{u_1}\sigma _{u_2}$, and $\varvec{\nu }\sim N({\textbf{0}}, \varvec{\varSigma }_\nu )$ where an MCAR correlation structure is imposed such that $\varvec{\varSigma }_\nu$ is defined in (2). To reduce the computational burdens in the MCAR structure (computing large covariance matrices and checking for positive-definiteness of covariance matrices in each iteration of the MCMC algorithm), and also to fit the MCAR($\alpha _1, \alpha _2, \varvec{\varSigma }_b$) efficiently via JAGS (Plummer 2003), we reparameterize $\varvec{\nu }$ as a linear combination of independent latent Gaussian processes via a $2\times 2$ full rank lower triangular matrix ${\textbf{A}}$, as proposed in Jin et al. (2007). More specifically, $\varvec{\nu }= ({\textbf{A}}\otimes {\textbf{I}}) {\textbf{f}}$, where ${\textbf{I}}$ is an $n \times n$ identity matrix, ${\textbf{f}}= ({\textbf{f}}_1^\textrm{T}, {\textbf{f}}_2^\textrm{T})^\textrm{T}$, and each ${\textbf{f}}_\ell$ ($\ell = 1,2$) is an $n\times 1$ independent latent Gaussian process with a conditional autoregressive (CAR) correlation structure, i.e., ${\textbf{f}}_\ell \sim {\mathcal {N}} \lbrace {\textbf{0}}, ({\textbf{D}}-\alpha _\ell {\textbf{W}})^{-1} \rbrace$ with ${\textbf{D}}$, ${\textbf{W}}$ and $\alpha _\ell$ defined as in Sect. 2.1. Under this representation, the covariance of $\varvec{\nu }$ in (2) can be rewritten as

$$\begin{aligned} \varvec{\varSigma }_\nu= & \left( {\textbf{A}}\otimes {\textbf{I}}\right) \text {Bdiag} \left\{ ({\varvec{\varSigma }}_{w})_{1}, ({\varvec{\varSigma }}_{w})_{2}\right\} \left( {\textbf{A}}^\textrm{T}\otimes {\textbf{I}}\right) \\= & \text {Bdiag} \left\{ ({\widetilde{\varvec{\varSigma }}}_{w})_{1}, ({\widetilde{\varvec{\varSigma }}}_{w})_{2} \right\} \left( {\textbf{A}}{\textbf{A}}^\textrm{T}\otimes {\textbf{I}}\right) \text {Bdiag} \left\{ ({\widetilde{\varvec{\varSigma }}}_{w})_{1}^\textrm{T}, ({\widetilde{\varvec{\varSigma }}}_{w})_{2}^\textrm{T}\right\} \\\equiv & \text {Bdiag} \left\{ ({\widetilde{\varvec{\varSigma }}}_{w})_{1}, ({\widetilde{\varvec{\varSigma }}}_{w})_{2}\right\} \left( \varvec{\varSigma }_b \otimes {\textbf{I}}\right) \text {Bdiag} \left\{ ({\widetilde{\varvec{\varSigma }}}_{w})_{1}^\textrm{T}, ({\widetilde{\varvec{\varSigma }}}_{w})^\textrm{T}_{2}\right\} , \end{aligned}$$

where the between-outcome covariance matrix $\varvec{\varSigma }_b = {\textbf{A}}{\textbf{A}}^\textrm{T}$.

We utilize thin-plate splines in estimation of the time-varying coefficients in our submodels. Consider the following expansion of the time-varying coefficient functions onto penalized low-rank thin-plate spline bases,

$$\begin{aligned} & \beta _{* p}(t) = a_{* p0 } + a_{* p1} t + \sum _{r=1}^{R} \phi _{* pr} |t- \kappa _r|^3 \\ & \gamma _{* q}(t) = b_{* q0} + b_{* q1} t + \sum _{r=1}^{R} \psi _{* qr} |t- \kappa _r|^3, \end{aligned}$$

where $a_{* p0}, a_{* p1}, b_{* q0}, b_{* q1},\varvec{\phi }_{* p}= (\phi _{* p1}, \ldots , \phi _{* pR})^\textrm{T},$ and $\varvec{\psi }_{* q}= (\psi _{* q1}, \ldots , \psi _{* qR})^\textrm{T}$ are the expansion coefficients, $\kappa _1<\kappa _2<\ldots <\kappa _R$ are the fixed knots, $p=1,\ldots , P$, and $q=1, \ldots , Q$ ($*$ denoting *y* or *s*). We take $\kappa _r$ to be the sample quantile of the time points corresponding to probability $r/(R+1)$ and we consider a relatively large number of knots (between 5 and 20) to ensure the desired flexibility (Ruppert et al. 2003; Crainiceanu et al. 2005). To avoid overfitting and obtain sufficiently smooth fitted curves, we include a penalty matrix $\varvec{\varOmega }_R$ with $(r, r^\prime )$th entry $|\kappa _{r^\prime }- \kappa _r|^3$ penalizing the coefficients of $|t- \kappa _r|^3$. Specifically, the P-spline expansions are expressed as

6 $$\begin{aligned} & \beta _{* p}(t_k) = a_{* p0 } + a_{* p1} t_k + \sum _{r=1}^{R} {\tilde{\phi }}_{* pr} \,e_{kr} \nonumber \\ & \gamma _{* q}(t_k) = b_{* q0} + b_{* q1} t_k + \sum _{r=1}^{R}{\tilde{\psi }}_{* qr} \, e_{kr}, \end{aligned}$$

where ${\textbf{a}}_{* p} = (a_{* p 0}, a_{* p 1})$, ${\textbf{b}}_{* q} = (b_{* q 0}, b_{* q 1})$, $\varvec{{\tilde{\phi }}}_{* p} = ({\tilde{\phi }}_{* p1}, \ldots , {\tilde{\phi }}_{* pR})^\textrm{T}= \varvec{\varOmega }_R^{1/2} \varvec{\phi }_{* p}$ with $\text {cov}(\varvec{{\tilde{\phi }}}_{* p}) = \sigma ^2_{{\tilde{\phi }}_{* p}}{\textbf{I}}$, $\varvec{{\tilde{\psi }}}_{* q} = ({\tilde{\psi }}_{* q 1}, \ldots , {\tilde{\psi }}_{* q R})^\textrm{T}= \varvec{\varOmega }_R^{1/2} \varvec{\psi }_{* q}$ with $\text {cov}(\varvec{{\tilde{\psi }}}_{* q}) = \sigma ^2_{{\tilde{\psi }}_{* q}}{\textbf{I}}$, $e_{kr}$ denotes the (*k*, *r*)th entry of the transformation ${\mathcal {E}} = {\mathcal {E}}_R\varvec{\varOmega }_R^{-1/2}$ of the design matrix ${\mathcal {E}}_R$ with the *k*th row $\lbrace |t_k- \kappa _1|^3, \ldots , |t_k- \kappa _R|^3 \rbrace$, ${\textbf{I}}$ is an $R\times R$ identity matrix, and $k=1,\ldots , K$ (Crainiceanu et al. 2005). We model the baseline hazard function $h_0(t)$ using the same thin-plate splines approach such that $\log \lbrace h_0(t_k) \rbrace = c_{0} + c_{1} t_k + \sum _{r=1}^{R}{\tilde{\omega }}_{r} \, e_{kr}$ with ${\textbf{c}}_{h_0} = (c_{0}, c_{1})$ and $\varvec{{\tilde{\omega }}}_{h_0} = ({\tilde{\omega }}_{1}, \ldots , {\tilde{\omega }}_{R})$. Note that proposed estimation and inference procedures can also accommodate parametric and other nonparametric forms for the baseline hazard function.

Under the thin-plate splines approach, the posterior density in (3) is rewritten as

$$\begin{aligned} p (\varvec{\theta }_{{{\mathcal T}_{\mathcal P}}}, {\textbf{u}}_{ij}, \varvec{\nu }_{i} \mid {\textbf{Y}}_{ij}, T_{ij}, \delta _{ij})\propto & \prod _{k=1}^{n_{ij}} p(Y_{ijk} \mid {\textbf{u}}_{ij}, \varvec{\nu }_i, \varvec{\theta }_{{{\mathcal T}_{\mathcal P}}} )p(T_{ij}, \delta _{ij} \mid {\textbf{u}}_{ij}, \varvec{\nu }_{i}, \varvec{\theta }_{{{\mathcal T}_{\mathcal P}}}) \\ & \times \, p( {\textbf{u}}_{ij}, \varvec{\nu }_i\mid \varvec{\theta }_{{\mathcal T}_{\mathcal P}} ) p( \varvec{\theta }_{{\mathcal T}_{\mathcal P}} ), \end{aligned}$$

where $\varvec{\theta }_{{{\mathcal T}_{\mathcal P}}} = (\varvec{\theta }_{y,{{\mathcal T}_{\mathcal P}}}, \varvec{\theta }_{s,{{\mathcal T}_{\mathcal P}}}, \varvec{\theta }_{RE})^\textrm{T}$ is the vector of parameters including the coefficients from the thin-plate spline expansions $\varvec{\theta }_{y,{{\mathcal T}_{\mathcal P}}} = ({\textbf{a}}_y, {\textbf{b}}_y, \varvec{{\tilde{\phi }}}_y, \varvec{{\tilde{\psi }}}_y)$ and $\varvec{\theta }_{s,{{\mathcal T}_{\mathcal P}}} = ({\textbf{a}}_s, {\textbf{b}}_s, {\textbf{c}}_{h_0}, \varvec{{\tilde{\phi }}}_s, \varvec{{\tilde{\psi }}}_s, \varvec{{\tilde{\omega }}}_{h_0})$ with ${\textbf{a}}_{*} = ({\textbf{a}}_{* 1}, \ldots , {\textbf{a}}_{* P})$, ${\textbf{b}}_{*} = ({\textbf{b}}_{* 1}, \ldots , {\textbf{b}}_{* Q})$, $\varvec{{\tilde{\phi }}}_{*} = (\varvec{{\tilde{\phi }}}_{* 1}^\textrm{T}, \ldots ,$ $\varvec{{\tilde{\phi }}}_{* P}^\textrm{T})$, and $\varvec{{\tilde{\psi }}}_{*} = (\varvec{{\tilde{\psi }}}_{* 1}^\textrm{T}, \ldots , \varvec{{\tilde{\psi }}}^\textrm{T}_{* Q})$, and the parameters associated with REs $\varvec{\theta }_{RE} = (\sigma ^2_{u_1},$ $\sigma ^2_{u_2}, \rho _u, \alpha _1, \alpha _2, \varvec{\theta }_{\varSigma _b})$.

In terms of prior distributions, we use the normal distribution for each spline coefficient (6) such that $a_{* p 0} \sim N (0, \sigma ^2_{a_{* p0}})$, $a_{* p 1} \sim N (0, \sigma ^2_{a_{* p1}})$, $b_{* q 0} \sim N (0, \sigma ^2_{b_{* q0}})$, $b_{* q 1} \sim N (0, \sigma ^2_{b_{* q1}})$, ${\tilde{\phi }}_{* pr} \sim N (0, \sigma ^2_{{\tilde{\phi }}_{* p}})$, and ${\tilde{\psi }}_{* qr} \sim N(0, \sigma ^2_{{\tilde{\psi }}_{* q}})$ and for the variance components, we employ gamma priors $\sigma ^{-2}_{{\tilde{\phi }}_{* p}} \sim G (\lambda ^{(1)}_{{\tilde{\phi }}_{* p}}, \lambda ^{(2)}_{{\tilde{\phi }}_{* p}})$ and $\sigma ^{-2}_{{\tilde{\psi }}_{* q}} \sim G (\lambda ^{(1)}_{{\tilde{\psi }}_{* p}}, \lambda ^{(2)}_{{\tilde{\psi }}_{* p}})$, $p=1,\ldots , P$, $q=1,\ldots , Q$, and $r=1,\ldots , R$. Similar priors are utilized for the spline coefficients used in the estimation of the baseline hazard function. The common practice is to set the variance terms in the normal priors to be very large, e.g., $10^6$. In the Gamma priors for the hyperparameters, Crainiceanu et al. (2005) suggested choosing the parameters such that the mean is 1 and the variance is very large, e.g., $(\lambda ^{(1)}, \lambda ^{(2)}) = (10^{-6}, 10^{-6})$. However, alternative specifications are also available depending on the smoothness of the function that is approximated via the thin-plate approach and it is highly recommended to inspect the results under a number of different choices since the estimates of the variance components are known to be sensitive to the prior specification (Crainiceanu et al. 2005; Gelman 2006). For the variance terms associated with the subject-level REs, we assume Gamma priors $\sigma ^{-2}_{u_1} \sim G (\lambda ^{(1)}_{u_1}, \lambda ^{(2)}_{u_1})$ and $\sigma ^{-2}_{u_2} \sim G(\lambda ^{(1)}_{u_2}, \lambda ^{(2)}_{u_2})$. Uniform priors are utilized for $\rho _u$, the correlation between the subject-level REs in longitudinal and survival submodels, and the spatial smoothing parameters $(\alpha _1, \alpha _2)$. For the between-outcome matrix in the MCAR model for the region-level REs, $\varvec{\varSigma }_b = {\textbf{A}}{\textbf{A}}^\textrm{T}$, elementwise priors are imposed on the lower triangular matrix ${\textbf{A}}$, $a_{\ell \ell } \sim$ Lognormal$(\mu _{a_{\ell \ell }}, \sigma ^2_{a_{\ell \ell }})$, $\ell =1, 2,$ and $a_{\ell \ell ^\prime } \sim N(\mu _{a_{\ell \ell ^\prime }}, \sigma ^2_{a_{\ell \ell ^\prime }})$ for $\ell \ne \ell ^\prime$.

For inference on the varying-coefficient functions, we use pointwise and simultaneous credible intervals (Crainiceanu et al. 2007). Let *f*(*t*) denote a single varying-coefficient function (taken to be $h_0(t)$ or a varying coefficient function from the longitudinal or survival submodels, $\beta _{*}(t)$ or $\gamma _{*}(t)$, respectively) observed at time points $t_k$, for $k=1,\ldots , K$. Let ${\hat{f}}(t)$ and $\text {SD}\{f(t)\}$ denote the mean and standard deviation of *f*(*t*) obtained based on a total of *L* MCMC samples, respectively. Then the $(1-\alpha )$ pointwise credible intervals are given by $\left[ {\hat{f}}(t_k) \pm \varPhi _{\alpha /2} \text {SD} \{f(t_k)\}\right]$, where $\varPhi _{\alpha /2}$ denotes the $100\times (1-\alpha /2)$-percentile of the standard normal distribution. For the simultaneous credible intervals, let $c_{\alpha }$ be the $(1-\alpha )$ sample quantile of $\text {max}_{k=1,\ldots , K} |f^{(\ell )} (t_k) - {\hat{f}}(t_k)|/\text {SD}\{f(t_k)\}|$ with $f^{(\ell )} (t)$, $\ell =1\ldots , L$, denoting the $\ell$th MCMC sample. Then the $(1-\alpha )$ simultaneous credible interval for *f*(*t*) is given as $\left[ {\hat{f}}(t_k) \pm c_{\alpha } \text {SD} \{f(t_k)\}\right]$.

We note that, for simplicity of exposition, we describe the submodels using a common set of subject-and region-level time-invariant predictors; however, the estimation and inference procedures can accommodate (1) design vectors with different dimensionality and composition, and (2) both baseline and time-varying covariates, provided that the time-varying covariates in the survival submodel are exogenous adhering to the requirements of a proper Cox model ( Rizopoulos 2012, Section 3.4). Also, in the estimation procedure, we use the same number of knots *R* in each spline expansion, however, our estimation procedure can handle a different number of knots for each function.

All computations have been performed in R (version 4.0.2), and as there are no closed-form solutions for the posterior distributions, we fit our model using the Bayesian software JAGS (version 4.3.0) via the rjags package (Plummer et al. 2019).

## Simulation studies

### Design

We conducted simulation studies to assess the finite sample performance of the proposed estimation and inference procedures. For these studies, datasets were generated with similar characteristics to the USRDS data under varying number of regions. The maximum number of repeated measurements per subject was 20, mimicking measurements observed every three months for a maximum of 5 years of follow-up in USRDS. The repeated measurements were equally spaced on the interval [0, 1] before censoring by survival.

The subject- and region-level covariates were simulated from the normal distribution such that $X_{ij} \sim N(1, 0.5)$ and $Z_{i} \sim N(1.5, 1)$. The time-varying coefficient functions are set as $\varvec{\beta }_y(t) = \lbrace \beta _{y_0}(t), \beta _{y_1}(t) \rbrace ^\textrm{T}= { \lbrace \cos (3/2\pi t) - 1}$, ${\sin (2\pi t -1/8)\rbrace }^\textrm{T}$, $\gamma _y(t)=\cos (\pi t{-}0.5)$, $\beta _{s}(t) = \cos (2\pi t)$, and $\gamma _s(t) = \sin (3/4\pi t)$. The baseline hazard $h_0(t)$ was generated using the Weibull function with the parameter $\theta _{h_0} = 1.7$. The subject-level REs were simulated from a normal distribution such that ${\textbf{u}}_{ij} = (u_{1ij}, u_{2ij})^\textrm{T}\sim N( {\textbf{0}}, \varvec{\varSigma }_u)$ with $\varvec{\varSigma }_u = \left[ \sigma _{u_1}^2, \sigma _{u}; \sigma _{u},\sigma _{u_2}^2 \right]$, $\sigma _u = \rho _u \sigma _{u_1}\sigma _{u_2}$, $\sigma _{u_1}^2 = 1.5$, $\sigma _{u_2}^2 = 2.5$, and $\rho _u = 0.8$. The region-level REs $\varvec{\nu }$ are imposed an MCAR ($\alpha _1, \alpha _2, \varvec{\varSigma }_b$) structure, where $\varvec{\nu }= (\varvec{\nu }_{1}^\textrm{T}, \varvec{\nu }_{2}^\textrm{T})^\textrm{T}\sim N (\varvec{0}, \varvec{\varSigma }_\nu )$ as described in Sect. 2.1. More specifically, the between-outcome matrix $\varvec{\varSigma }_b$, a $2\times 2$ symmetric positive-definite matrix, is specified as $\left[ 0.40, 0.04; 0.04, 0.10\right]$ and the within-outcome matrix $(\varvec{\varSigma }_{w})_\ell$ is set as $(\varvec{\varSigma }_{w})_\ell =({\textbf{D}}-\alpha _\ell {\textbf{W}})^{-1}$, $\ell =1,2$, where ${\textbf{W}}$ and ${\textbf{D}}$ are specified using the map of $n = 49$ states in the contiguous U.S. (including the District of Columbia) and the map of $n = 476$ HSAs from our data application (Supplementary Figure 1), and $\alpha _\ell$ are set as $\alpha _1 = 0.3$ and $\alpha _2=0.2$. Then $\varvec{\varSigma }_\nu$ can be obtained via

$$\begin{aligned} \varvec{\varSigma }_\nu= & \text {Bdiag} \left\{ ({\widetilde{\varvec{\varSigma }}}_{w})_{1}, ({\widetilde{\varvec{\varSigma }}}_{w})_{2}\right\} \left( \varvec{\varSigma }_b \otimes {\textbf{I}}\right) \text {Bdiag} \left\{ ({\widetilde{\varvec{\varSigma }}}_{w})_{1}^\textrm{T}, ({\widetilde{\varvec{\varSigma }}}_{w})^\textrm{T}_{2}\right\} \\= & ({\widetilde{\varvec{\varSigma }}}_b \otimes I_n) \text {Bdiag}\left\{ (\varvec{\varSigma }_w)_1,(\varvec{\varSigma }_w)_2 \right\} ({\widetilde{\varvec{\varSigma }}}^\textrm{T}_b \otimes I_n), \end{aligned}$$

where $({\varvec{\varSigma }}_{w})_{\ell } = ({\widetilde{\varvec{\varSigma }}}_{w})_{\ell } ({\widetilde{\varvec{\varSigma }}}_{w})_{\ell }^\textrm{T}$, ${\varvec{\varSigma }}_{b} = {\widetilde{\varvec{\varSigma }}}_{b} {\widetilde{\varvec{\varSigma }}}_{b}^\textrm{T}$ via the Cholesky decomposition, and a tilde on any symmetric positive-definite matrix denotes the operator that returns the lower triangular matrix of the Cholesky decomposition. For each setting with $n=49$ and $n=476$ regions, 150 Monte Carlo datasets were generated.

The longitudinal outcome $Y_{ij}(\cdot )$ was simulated using an underlying normal latent variable $Y_{ij}^*(\cdot )$ such that $Y_{ij}(\cdot ) = I\{Y_{ij}^*(\cdot )>0\}$, and the mean of $Y_{ij}^*(\cdot )$ was determined by the longitudinal submodel given in (1). For the survival outcome, the true event times, $T_{ij}^*$, were simulated using the inverse probability integral transformation with a Weibull baseline hazard function (Bender et al. 2005). As described in Sect. 2.1, the observed time and the event indicator were calculated as $T_{ij}$ = $\text {min}(C_{ij}, T_{ij}^*)$ and $\delta _{ij} = I(T_{ij}\le C_{ij})$, respectively. Under this set-up, similar to the USRDS data, the overall hospitalization and censoring rates were approximately 29% and 62%, respectively.

### Simulation results

We estimated all time-varying coefficient functions and the baseline hazard function using the penalized thin-plate splines approach described in Sect. 2.2 with $R=10$ knots. In terms of priors, we set a large value for the variance terms ($\sigma ^2_{a_{* p0}}, \sigma ^2_{a_{* p1}}, \sigma ^2_{b_{* q0}}$, and $\sigma ^2_{b_{* q1}}$) in the normal priors for the spline coefficients such that $a_{* p 0} \sim N (0, 10^6)$, $a_{* p 1} \sim N (0, 10^6)$, $b_{* q 0} \sim N (0, 10^6)$, $b_{* q 1} \sim N (0, 10^6)$. To determine the parameters in the Gamma priors for the variance terms $\sigma ^2_{{\tilde{\phi }}_{* p}}$ and $\sigma ^2_{{\tilde{\psi }}_{* q}}$, we examined the estimated results under several different prior choices and chose $(\lambda ^{(1)}_{{\tilde{\phi }}_{yp}}, \lambda ^{(2)}_{{\tilde{\phi }}_{y p}}) = (\lambda ^{(1)}_{{\tilde{\psi }}_{y p}}, \lambda ^{(2)}_{{\tilde{\psi }}_{y p}}) = (10^{-6}, 10^{-6})$ and $(\lambda ^{(1)}_{{\tilde{\phi }}_{sp}}, \lambda ^{(2)}_{{\tilde{\phi }}_{sp}}) = (\lambda ^{(1)}_{{\tilde{\psi }}_{sp}}, \lambda ^{(2)}_{{\tilde{\psi }}_{sp}}) = (10^{-6}, 10^{-6})$ for the longitudinal and survival submodels, respectively. For the variance terms associated with the subject-level REs, we set $(\lambda ^{(1)}_{u_1}, \lambda ^{(2)}_{u_1}) = (2, 0.05)$ and $(\lambda ^{(1)}_{u_2}, \lambda ^{(2)}_{u_2}) = (2, 0.1)$. We put uniform priors (*U*(0.1, 1)) on the correlation $\rho _u$ and the spatial smoothing parameters $(\alpha _1, \alpha _2)$. In the MCAR model, for the between-outcome matrix, $\varvec{\varSigma }_b = {\textbf{A}}{\textbf{A}}^\textrm{T}$, we assigned elementwise priors on the lower triangular matrix ${\textbf{A}}$ such that $a_{\ell \ell } \sim$ Lognormal$(\mu _{a_{\ell \ell }}, \sigma ^2_{a_{\ell \ell }})$, $\ell =1, 2,$ and $a_{\ell \ell ^\prime } \sim N(\mu _{a_{\ell \ell ^\prime }}, \sigma ^2_{a_{\ell \ell ^\prime }})$ for $\ell \ne \ell ^\prime$. We performed our estimation procedure under different combinations of parameters in these priors and observed that the results are robust to these choices in our study. We set $(\mu _{a_{\ell \ell }}, \sigma ^2_{a_{\ell \ell }})=(0, 10)$ and $(\mu _{a_{\ell \ell ^\prime }}, \sigma ^2_{a_{\ell \ell ^\prime }}) = (0.5, 0.005)$ in the priors for the diagonal and off-diagonal terms, respectively.

We evaluate the performance of the proposed procedure in estimating the time-invariant ($\varvec{\theta }_{RE} = (\sigma ^2_{u_1}, \sigma ^2_{u_2}, \rho _u, \alpha _1, \alpha _2, \varvec{\theta }_{\varSigma _b})$ with $\varvec{\theta }_{\varSigma _b}$ denoting parameters associated with the between-outcome covariance matrix) and time-varying ($h_0(t)$ and varying-coefficient functions $\varvec{\beta }(t)$ or $\varvec{\gamma }(t)$) coefficients using the mean squared error (MSE) and root average squared error (RASE), respectively. RASE (Cai et al. 2000; Kürüm et al. 2014, 2016), a commonly used measure to examine the accuracy of varying-coefficient estimations, is defined as

7 $$\begin{aligned} RASE_{{\hat{f}}} = \left[ \dfrac{1}{K} \sum _{k=1}^{K} \left\{ \dfrac{f(t_k) -{\hat{f}}(t_k)}{\text {range} f(\cdot )} \right\} ^2 \right] ^{1/2}, \end{aligned}$$

where $K = 20$ and $f(\cdot )$ denotes a single varying-coefficient function.

**Fig. 1:**
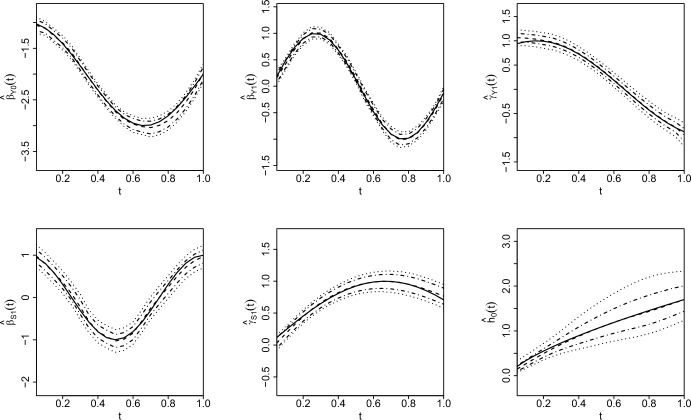
Estimated time-varying coefficient functions (dashed) in the longitudinal submodel (top row) and survival submodel (bottom row) from the simulation runs with median RASE among 150 Monte Carlo runs for $n = 476$ regions overlaying the true functions (solid) along with 95% simultaneous (dotted) and pointwise (dashed-dotted) credible intervals

The estimated time-varying coefficient functions in longitudinal and survival submodels are presented in Fig. 1, along with their simultaneous and pointwise credible intervals from the simulation runs with the median RASE based on $n=476$ regions, similar to the USRDS data. (Results for the case of $n=49$ regions are described in the supplementary materials in more detail.) These results indicate that the proposed method performed well in the simulation studies. More specifically, the estimates (dashed) track the true functions (solid) closely and mostly capture them well within the simultaneous (dotted) and pointwise (dashed-dotted) credible intervals for all varying-coefficient functions. As described in the supplementary materials (Supplementary Figure 2), the estimation and credible intervals of the varying-coefficient functions are improved (e.g., smaller bias and narrower pointwise and simultaneous credible intervals) with increasing number of regions ($n=476$ versus $n=49$ regions), as expected. This is also quantified in Table 1 which displays the median RASE values for the varying-coefficient functions along with average coverage probabilities of the 95% simultaneous and pointwise credible intervals. In particular, we observe that the median RASE values get smaller and average coverage probabilities increase as the number of regions increase.

**Fig. 2:**
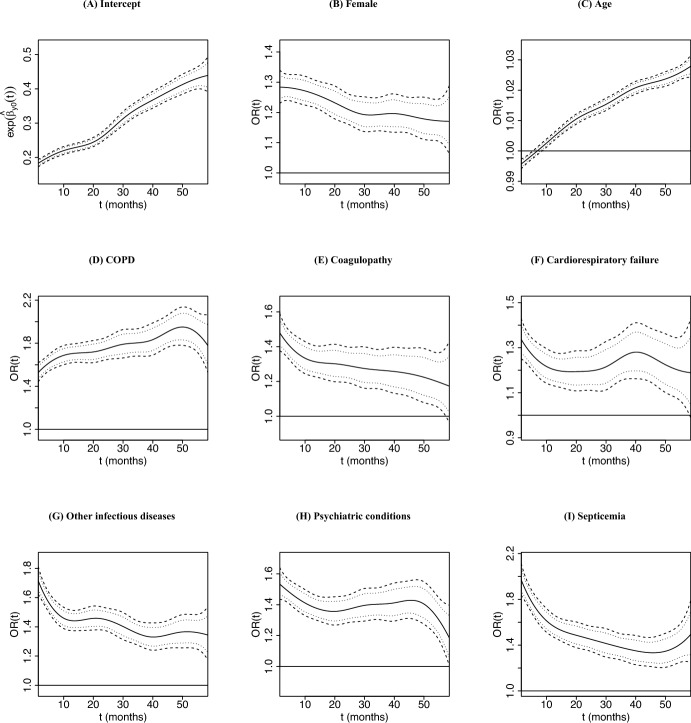
Estimated patient-level effects on hospitalization, time-varying odds ratio $OR(t) = \exp \lbrace {\hat{\beta }}_{y_*}(t) \rbrace$, (solid) along with their 95% simultaneous (dashed) and pointwise (dotted) credible intervals

Note that due to the clear boundary effects, that is, under-coverage at the boundaries, we report average coverage probabilities based on both simultaneous and pointwise credible bands for the time interval (0.2, 0.8) (Table 1). The coverage probabilities in this interval, which accounts for the boundary effects, are above the nominal level. These results are consistent with literature (Cox 1993; Krivobokova et al. 2010) and shows that the Bayesian credible intervals are more conservative. The estimated values for the time-invariant parameters $(\sigma ^2_{u_1}, \sigma ^2_{u_2}, \rho _u, \alpha _1, \alpha _2)$ along with their corresponding 95% pointwise credible intervals for $n=49$ and $n=476$ are given in Table 1. Similar to results on the varying-coefficient functions, our method targets the true values closely for the time-invariant parameters. As expected, as the number of regions increases, the bias gets smaller, and the pointwise credible intervals get narrower.

**Table 1:** Simulation results for time-varying and time-invariant estimates under $n = 49$ and $n=476$ based on 150 datasets. (A) For time-varying estimates, given are the median root average squared error (RASE) along with average coverage probabilities of the 95% simultaneous ($\hbox {CP}_\text {S}$) and pointwise (CP) credible intervals (computed for the time interval (0.2, 0.8)), and (B) for time-invariant estimates, given are bias, mean squared error (MSE), and average coverage probabilities 95% pointwise (CP) credible intervals

	Number of regions, $n = 49$			Number of regions, $n = 476$		
**(A)**	RASE	$\hbox {CP}_\text {S}$(%)	CP(%)	RASE	$\hbox {CP}_\text {S}$(%)	CP(%)
Longitudinal						
$\beta _{y0}(t)$	0.066	95.8	95.3	0.049	96.4	95.8
$\beta _{y1}(t)$	0.055	96.1	95.1	0.040	96.9	95.5
$\gamma _{y}(t)$	0.053	97.0	96.5	0.031	97.1	96.8
Survival						
$\beta _{s}(t)$	0.055	96.5	95.3	0.041	96.8	95.7
$\gamma _{s}(t)$	0.065	96.4	95.1	0.039	96.6	95.6
$h_0(t)$	0.083	96.9	95.6	0.058	97.0	95.9

**(B)**	Bias	MSE	CP(%)	Bias	MSE	CP(%)
$\sigma _{u_1}^2$	0.027	0.004	94.9	0.020	0.002	95.4
$\sigma _{u_2}^2$	$-0.051$	0.036	94.0	$-0.028$	0.015	95.0
$\rho _u$	0.005	0.002	93.9	0.003	0.002	95.9
$\alpha _1$	$-0.049$	0.024	90.1	$-0.035$	0.014	93.9
$\alpha _2$	$-0.035$	0.028	89.7	$-0.028$	0.011	93.0

## Data analysis

### USRDS data: study cohort, patient characteristics, and region-level risk factors

The USRDS is a national database that collects data on nearly all U.S. patients with ESKD on dialysis. Our study cohort included patients of age 18 years or older who transitioned to dialysis between January 1, 2006 and December 31, 2008. The observation period started from day 91 of dialysis, following the USRDS Researcher’s Guide “90-day rule” recommendation to allow for completion of the Medicare eligibility application process and establishment of stable dialysis treatment modality (United States Renal Data System 2014). The maximum follow-up period for the patients was 5 years, with the last follow-up date as December 31, 2013, where follow-up was truncated if a patient switched dialysis facilities. Consistent with the national annual USRDS reporting, we considered HSA as the unit for region. In order to avoid instability in the estimation, we merged HSAs to guarantee that each region contained at least 50 patients. (This reduced the number of HSAs from 649 to 476 used in the analyisis.) The final study cohort had 1,018,334 observations over time on 121,839 patients across 476 regions/HSAs. The overall hospitalization rate was 27.1% and the censoring was 64.0%.

The overall average age of the study cohort was 65 years (SD 15), and 45% of the patients were female. Common baseline comorbidities included chronic obstructive pulmonary disease (COPD; 19.4%), septicemia (10.5%), other infectious diseases (24.3%), cardiorespiratory failure (12.6%), coagulopathy (8.6%), and psychiatric conditions (11.9%). The median length of patient follow-up was 18 months (Q1–Q3: 3–42), and the mean number of hospitalizations was 1.8 per person-year (SD 2.2). The median marginal (unadjusted) survival was 4.1 years.

The proposed STM-JM was employed to study the time-varying effects of patient-level and region-level covariates on both longitudinal and survival outcomes. Patient-level covariates included age (mean-centered), sex, and baseline comorbidities (COPD, coagulopathy, cardiorespiratory failure, septicemia, other infectious diseases/pneu-monias, and psychiatric disorders). Region-level covariates in our analysis included urbanicity, area deprivation index (ADI), and medical underservice index (MUI) in both submodels. To capture urbanicity, each HSA was categorized into three classes: large metropolitan, small metropolitan, or non-metropolitan regions. The category of each HSA was determined according to the class that the majority of the counties within the HSA fall into, assigned by the urban-rural classification scheme from the National Center for Health Statistics (https://www.cdc.gov/nchs/data_access/urban_rural.htm). Non-metropolitan regions were taken as the reference group. The second region-level covariate, ADI, captures the socioeconomic status of HSAs, consisting of 17 education, employment, housing-quality, and poverty measures (Kind and Buckingham 2018). The rank-based index takes values between 0 and 100 (higher values correspond to lower socioeconomic status and higher deprivation) and is available at the census block group level (available at https://www.neighborhoodatlas. medicine.wisc.edu). ADIs were averaged over census block groups within each HSA to obtain HSA-level indices. MUI reflects the medical service availability within each HSA and takes on values between 0 and 1, with higher indices corresponding to higher levels of medically underservice. The index is released by the Health Resources and Services Administration at the census tract/county subdivision level and is available at https://data.hrsa.gov/tools/shortage-area/, where medically underserved tracts/subdivisions are areas with too few primary care providers, high infant mortality, high poverty or a high elderly population. The proportion of census tracts/county subdivisions designated as underserved was first targeted for each county, and county MUIs were then averaged within each HSA to derive the HSA-level MUI index. The USRDS data was linked to the aforementioned data sources for region-level information via zip code. The covariates ADI and MUI were mean-centered for ease of interpretation in modeling.

### Results

Under the Bayesian estimation discussed in Sect. 2.2, posterior samples were obtained by running three parallel chains with adaptation phase (5000 iterations) and burn-in period (1500 iterations). The thinning was selected to keep 1500 posterior samples in each chain; thus, 4500 samples were used for estimation and inference. Trace plots are provided in the supplementary materials (Figure S3). In addition to examining the trace plots for convergence, we also monitored the scale reduction factor, R, as recommended by Gelman and Rubin (1992), and ensured that R $\approx$ 1. In terms of prior choices, we employed the same distributions and parameters as described in the simulation study. The time-varying coefficients for the patient- and region-level covariates were estimated using the thin-plate splines approach with 10 knots located at the sample quantiles of the 5-year follow-up time period. The subject-level RE variances were estimated as ${\hat{\sigma }}_{u_1}^2 = 1.718$ (SE 0.014) and ${\hat{\sigma }}^2_{u_2} = 3.007$ (SE 0.179) The estimated correlation between the subject-level REs was ${\hat{\rho }}_u = 0.917$ (SE 0.009), confirming a significant association between hospitalization and mortality, as expected. The spatial smoothing parameters corresponding to each outcome were estimated as ${\hat{\alpha }}_1 = 0.314$ (SE 0.143) and ${\hat{\alpha }}_2 = 0.292$ (SE 0.129). After adjusting for subject-level REs, the remaining correlation between the responses that is carried by the region-level REs was estimated to be 0.207 (SE 0.083), using $\varSigma _b$, indicating that there is still a significant association remaining between the outcomes.

The effects of patient-level risk factors on longitudinal hospitalizations are presented in Fig. 2 (solid line), along with 95% simultaneous (dashed lines) and pointwise credible intervals (dotted lines). The estimated intercept ${\hat{\beta }}_{y0}(t)$ in the longitudinal submodel, which represents the odds of hospitalization $\exp \lbrace {\hat{\beta }}_{y0}(t)\rbrace$ for male patients initiating dialysis at mean age 65 with no comorbidities within non-metropolitan region (reference group) with average ADI and MUI, is increasing over time after transition to dialysis (Fig. 2A). Females have higher odds of hospitalization, but the magnitude of the difference declines slightly during the 5-year follow-up (Fig. 2B). Older age at the initiation of dialysis is associated with increasingly higher odds of hospitalization starting at about 12 months (i.e., after the fragile first year post-dialysis transition; Fig. 2C).

During the 5-year study follow-up, all comorbidities were found to be associated with significantly higher odds of hospitalization (Fig. 2D–I). More specifically, the effect of COPD increases as the patients remain longer on dialysis, with the highest risk observed towards the end of the 5-year follow-up (month 49): OR$(t) = \exp \lbrace {\hat{\beta }}_{y3}(t = 50 \; \text {month})\rbrace \sim 1.951$. The effects of acute comorbidities, such as septicemia and other infectious diseases/pneumonias, decrease as patients stay longer on dialysis, with the highest risk period observed during the first 12 months on dialysis (OR(*t*) ranges from 1.570 to 1.968 and 1.444 to 1.713 for septicemia and other infectious diseases/pneumonia, respectively). Similarly, coagulopathy and psychiatric conditions are associated with significantly higher risk of hospitalization, with the highest risks estimated during the first year post-dialysis transition, and their effects remain relatively stable after this period. For patients with cardiorespiratory failure, we observe that the effect remains mostly constant after the first 12 months on dialysis, except for a slight increase in the estimated effect around month 40.

**Fig. 3:**
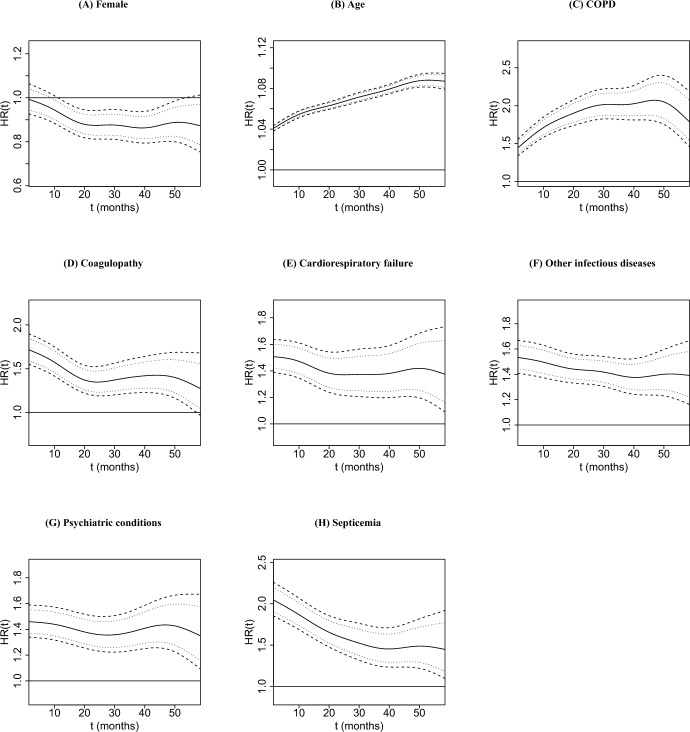
Estimated patient-level effects on survival, time-varying hazard ratios $HR(t) = \exp \lbrace {\hat{\beta }}_{s_*}(t) \rbrace$, (solid) along with their 95% simultaneous (dashed) and pointwise (dotted) credible intervals

Next, we examine the time-varying effects of patient-level covariates on the hazard of death displayed in Fig. 3. Females have a lower hazard of death, starting at about 12 months (Fig. 3A). As expected, older age at dialysis transition is associated with an increased hazard of death (Fig. 3B). Similar to their effects on hospitalization, all comorbidities are also associated with significantly higher hazard of death during the 5-year follow-up (Fig. 3C–H). In particular, the effects of coagulopathy and septicemia are the largest during the first 24 months post-dialysis transition, where OR(*t*) ranges from 1.348 to 1.716 and 1.593 to 2.046 for coagulopathy and septicemia, respectively, and we observe a declining trend in both effects for longer follow-up times. The effect of COPD increases during the first two years post-dialysis transition and is mostly stable after this time period. We observe similar patterns in the effects of psychiatric conditions, cardiorespiratory failure, and other infectious diseases/pneumonias, where they are significant and remain mostly constant over the 5-year follow-up period.

**Fig. 4:**
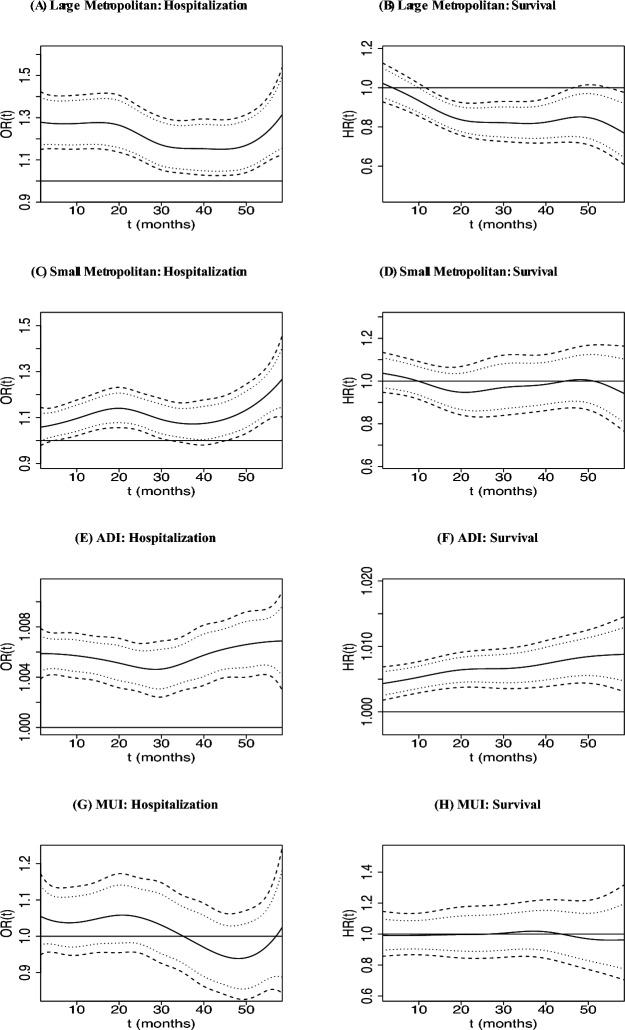
Estimated region-level effects for large and small metropolitan regions (non-metropolitan regions were taken as the reference group), area deprivation index (ADI) (centered) and medical underservice index (MUI) (centered) on hospitalization (**A**, **C**, **E**, and **G**), time-varying odds ratio $OR(t) = \exp \lbrace {\hat{\gamma }}_{y_*}(t) \rbrace$, and on survival (**B**, **D**, **F**, and **H**) time-varying hazard ratios $HR(t) = \exp \lbrace {\hat{\gamma }}_{s_*}(t) \rbrace$, (solid) along with their 95% simultaneous (dashed) and pointwise (dotted) credible intervals

At the region level, the estimated time-varying effects of risk factors are displayed in Fig. 4. We observe that large metropolitan regions have higher hospitalization rates than non-metropolitan regions (reference group), especially at the initiation of dialysis (Fig. 4A). For mortality, the difference between large metropolitan and non-metropolitan regions becomes significant after the first year on dialysis, where large metropolitan regions have a lower hazard of death (Fig. 4B). Small metropolitan regions mostly have significantly higher hospitalization rates than non-metropolitan regions, whereas we found no significant difference in mortality risk Fig. 4C, D). Higher levels of ADI is associated with both significantly higher odds hospitalization and hazard of death (Fig. 4E, F). The effect of MUI on hospitalizations and mortality are not significant as the credible intervals include zero (Fig. 4G, H). We caution the reader that above results focus on understanding the association between the outcomes and their corresponding subject- and region-level factors and should not be confused with inferential statements of a causal nature.

**Fig. 5:**
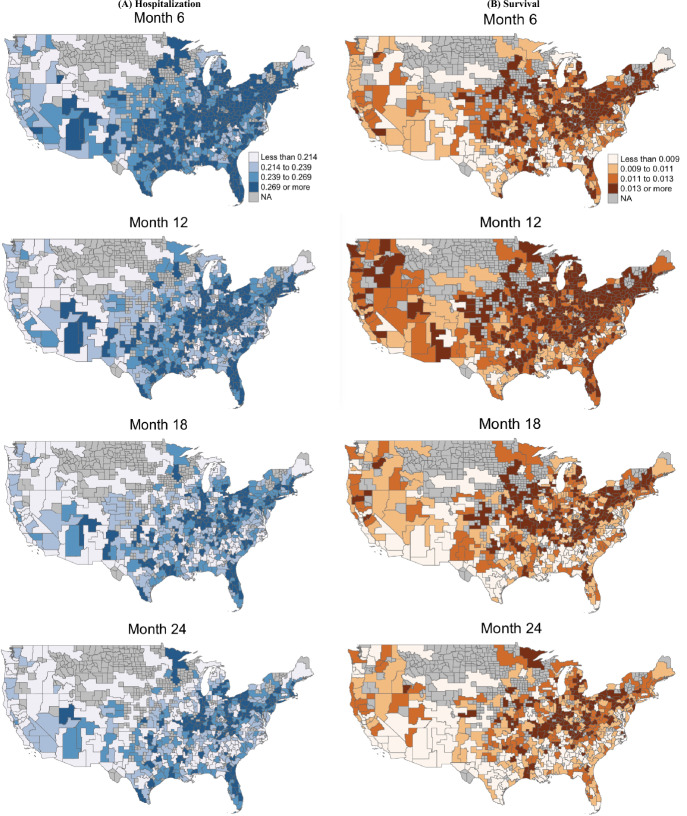
Estimated **A** hospitalization and **B** hazard probabilities from the 6th, 12th, 18th, and 24th months after transitioning to dialysis for all health service areas (regions). The region-specific predictions are averages of subject-specific predictions across all subjects that were alive at months 6, 12, 18, and 24 post-initiation of dialysis within each region (i.e., health service area)

To evaluate the spatial and temporal variations jointly in hospitalization and mortality risks while accounting for the time-varying effects of multilevel risk factors, Fig. 5 shows region-specific predictions from the joint model at 6, 12, 18, and 24 months after initiation of dialysis. The region-specific predictions were obtained via averaging subject-specific predictions across all patients that were alive at months 6, 12, 18, and 24 post-initiation of dialysis within each region (i.e., health service area). These subject-specific predictions at each time point were calculated utilizing the model fit and each patient’s corresponding covariate values. Overall, both sets of maps demonstrate elevated hospitalization and mortality risks in Massachusetts to southern Texas and Florida and a decreasing trend in both risks for longer follow-up times on dialysis. Across the U.S., we observe the highest risks for both outcomes in the northeast and central regions.

We observed more variation over time for hospitalization than mortality (see Fig. 5). Thus, we further examine the variation in hospitalization in Table 2, which includes the 25th and 75th percentiles and median values of the predicted region-specific hospitalization probabilities at month 6, 12, 18 and 24 for five major zones across the U.S.: West (54 HSAs), Midwest (124 HSAs), Southwest (60 HSAs), Southeast (186 HSAs), and Northeast (52 HSAs). Similar to the maps presented in Fig. 5A, the highest median predicted hospitalization probabilities are observed in the Northeast, followed by the Midwest. For example, after transitioning to dialysis at month 6, the median hospitalization risks in the Northeast (0.266) and Midwest (0.257) are 11.8% and 8.0% higher than the overall median (0.238) across all HSAs, respectively. In contrast, the West has the lowest median hospitalization probability at 0.197, which is about 17.2% lower than the overall median (0.238). A similar trend is noted across all time points (months 6, 12, 18, and 24). As we have observed in Fig. 5A, the predicted hospitalization probabilities decline over time. More specifically, the overall median hospitalization probability decreased from 0.238 at month 6 to 0.209 at month 24, which is nearly a 12.2% decline.

**Table 2:** The 25th, 50th and 75th percentiles of the predicted hospitalization probabilities for health service areas (HSAs) within five U.S. zones (West, Midwest, Southwest, Southeast and Northeast) and overall (all HSAs) at month 6, 12, 18, and 24 after transitioning to dialysis

Hospitalization probability (standard error)							
Month	Percentile	Overall	West	Midwest	Southwest	Southeast	Northeast
6	25	0.114 (0.001)	0.092 (0.001)	0.125 (0.001)	0.110 (0.001)	0.114 (0.001)	0.131 (0.002)
	50	0.238 (0.001)	0.197 (0.002)	0.257 (0.002)	0.230 (0.002)	0.237 (0.001)	0.266 (0.002)
	75	0.430 (0.001)	0.370 (0.003)	0.453 (0.003)	0.418 (0.003)	0.430 (0.002)	0.465 (0.003)
12	25	0.110 (0.001)	0.090 (0.001)	0.120 (0.001)	0.106 (0.001)	0.109 (0.001)	0.127 (0.002)
	50	0.223 (0.001)	0.187 (0.002)	0.240 (0.002)	0.216 (0.002)	0.222 (0.001)	0.251 (0.002)
	75	0.394 (0.001)	0.340 (0.003)	0.414 (0.003)	0.384 (0.003)	0.393 (0.002)	0.429 (0.003)
18	25	0.106 (0.001)	0.088 (0.001)	0.116 (0.001)	0.101 (0.001)	0.104 (0.001)	0.124 (0.001)
	50	0.211 (0.001)	0.179 (0.002)	0.227 (0.002)	0.203 (0.002)	0.209 (0.002)	0.242 (0.002)
	75	0.369 (0.002)	0.321 (0.003)	0.387 (0.003)	0.356 (0.003)	0.367 (0.003)	0.409 (0.003)
24	25	0.106 (0.001)	0.088 (0.001)	0.117 (0.001)	0.099 (0.001)	0.105 (0.001)	0.125 (0.001)
	50	0.209 (0.001)	0.178 (0.002)	0.225 (0.002)	0.198 (0.002)	0.206 (0.002)	0.239 (0.002)
	75	0.359 (0.001)	0.314 (0.003)	0.378 (0.003)	0.342 (0.003)	0.356 (0.002)	0.398 (0.003)

### Model fit

We examined the relative model fit using the deviance information criterion (DIC), a Bayesian measure of model fit and complexity that can conveniently be computed based on samples generated by the MCMC (See Spiegelhalter et al. 2002; Gelman et al. 2014). In particular, we compared DIC for STM-JM fit with two simpler models: (M1) includes time-varying regression coefficients and ignores the spatial dependency, and (M2) incorporates the spatial dependency through an MCAR correlation structure and includes time-invariant regression coefficients, that is, ignores temporal patterns. Table 3 summarizes the model fits via DIC showing that the DIC for the STM-JM is the smallest and, furthermore, the difference in DICs between each simpler model with the (full) STM-JM is substantial, clearly demonstrating that a better fit is provided by the STM-JM. Further details and specification of these models can be found in the Supplementary Materials (Online Appendix C).

**Table 3:** Bayesian model fit based on deviance information criterion (DIC) for the STM-JM compared to two simpler models (M1: only includes time-varying coefficients, ignores spatial dependency, M2: incorporates spatial dependency and time-invariant coefficients, that is, ignores temporal patterns)

Model	DIC	DIC difference with STM-JM
M1: only time-varying coefficients	24370436522	8974748
M2: only spatial dependency	24369986409	8524635
STM-JM: incorporates both spatial dependency and temporal patterns	24361461774	–

## Discussion

Motivated by the need to more fully elucidate the time-varying effects of risk factors on the correlated outcomes of longitudinal hospitalizations and survival in the U.S. dialysis population while incorporating key features of the USRDS data (three-level hierarchy and spatiotemporal variations), we developed a novel spatiotemporal multilevel joint model. This work addresses critical methodological gaps in the joint modeling literature. First, it proposes a joint modeling approach that (a) accommodates a three-level hierarchical data, (b) incorporates the spatiotemporal variations, and (c) allows for studying time-varying effects of multilevel risk factors at the individual and cluster (region) levels. Second, to our knowledge, this is the first spatiotemporal joint modeling framework for longitudinal and survival outcomes with time-varying coefficients for multilevel data.

Furthermore, the application of our proposed methodology to the USRDS data, where we modeled individual-level hospitalization and mortality risks, provides important insights. Our data analysis identified significant subject- and region-level risk factors for hospitalization and mortality and identified regions and specific time periods with high hospitalizations and mortality risks after transitioning to dialysis. For example, as discussed in Sect. 4.2, at the subject level, a history of acute comorbid conditions such as infectious diseases and septicemia prior to transitioning to dialysis is associated with a substantial risk of hospitalization and hazard of death, especially during the first year, and although the risk declines after the first year, it remains significantly high. Such information on the time-varying specificity of the effects of patient risk factors may potentially contribute to the development of patient monitoring/management procedure to reduce the risk of hospitalization.

At the region-level, large metropolitan areas and higher ADI levels were identified as significant risk factors associated with both hospitalization and mortality risks. In addition, our analysis of spatiotemporal variations identified a band of regions with higher hospitalization and mortality risks across the U.S. spanning Massachusetts to southern Texas and Florida in the early time periods after transitioning to dialysis. Future studies examining potential variation in practice patterns, policies, and resource allocation across regions are needed to elucidate the sources for significant spatial variation in hospitalization and mortality in the dialysis population.

Although previous analyses of the USRDS data have considered the spatiotemporal variations in hospitalization and mortality risks across the U.S., those studies utilized the aggregated rates of these outcomes (at the region or facility level). Our analysis based on subject-level data not only incorporated the multilevel risk factors more naturally than methods using aggregated data, it also yielded more precise estimates of the time-varying effects of the multilevel risk factors with narrower confidence intervals and presented a more targeted study in understanding the spatiotemporal variations of hospitalization and mortality risks across the U.S.

We note several areas for methodological extension in our current work. Firstly, while our utilization of varying coefficient models enabled us to discern between time-invariant and time-varying effects in the USRDS data application—a crucial distinction that would otherwise remain obscure—it may be of interest in subsequent modeling to explore mixture of covariate effects (fixed and time-varying). Such an approach would require semivarying modeling, which accommodates both time-varying and time-invariant coefficients. Developing the semivarying extension and its corresponding estimation procedure is important and warrants dedicated attention as future work.

Secondly, in our framework, we followed the shared random effects (subject- and region-level) approach to link the two outcomes. However, we recognize the importance of developing joint models and estimation procedures under alternative linking structures, such as incorporating the longitudinal outcome directly into the survival outcome with time-varying specifications. This presents a separate and exciting area for future research, one that is especially important in the development of dynamic prediction methods. It is crucial to note that developing such a prediction framework would pose unique challenges, particularly concerning the dependency of region-level random effects. Unlike existing dynamic prediction methods that rely on the independence of subject-level random effects, the dependency among region-level random effects complicates individual predictions. Finally, incorporation of random effects for regression coefficients may enhance the flexibility of the models. While conceptually possible, estimation under the joint modeling scheme is inherently intensive, and including more terms would increase this intensity. Therefore, before such extensions are explored, we suggest considering the trade-off between model flexibility and estimation intensity.

## Supplementary Information

Below is the link to the electronic supplementary material.

Supplementary file 1 (pdf 1374 KB)

## Data Availability

The release of the data used in this paper is governed by the National Institute of Diabetes and Digestive and Kidney Diseases through the USRDS Coordinating Center. The data can be requested from the USRDS through a data use agreement.
