# Supplementary material for: Spatiotemporal multilevel joint modeling of longitudinal and survival outcomes in end-stage kidney disease
Source: Lifetime Data Anal. 2024 Oct 4;30(4):827–52. doi: 10.1007/s10985-024-09635-w (PMC11502599; doi:10.1007/s10985-024-09635-w)
Supplement: Supplementary file 1 — (pdf 1374 KB) [file 10985_2024_9635_MOESM1_ESM.pdf]

Noname manuscript No.
(will be inserted by the editor)

Supplementary Materials for 'Spatiotemporal Multilevel Joint Modeling of Longitudinal and Survival Outcomes in End-Stage Kidney Disease'

the date of receipt and acceptance should be inserted later

Appendices

Online Appendix A. Details on the Simulation Study

This section provides further details on the simulation study set-up and the results from $n = 49$. The maps displayed in Figure S1 outlines the $n = 49$ states in the contiguous U.S. (including the District of Columbia) and $n = 476$ health services areas (HSAs: geographic regions with relatively self-contained infrastructure for the provision of hospital care). These maps are used to specify the spatial covariance $(\Sigma_w)_\ell = (\mathbf{D} - \alpha_\ell \mathbf{W})^{-1}$, in particular, $\mathbf{D}$ the diagonal matrix with diagonal elements d_i denoting the total number of neighbors of the ith region, and $\mathbf{W} = \{w_{ii'}\}$ the adjacency matrix that describes the neighborhood structure of the regions such that $w_{ii} = 0$ by convention, $w_{ii'} = 1$ if regions i and i' ($i \neq i'$) are neighbors, and $w_{ii'} = 0$ otherwise.

The estimated time-varying coefficient functions in longitudinal and survival submodels for $n = 49$ are given in Figure S2, along with their simultaneous and pointwise credible intervals from the simulation runs with the median RASE. We observe that overall our procedure performed well in this simulation setting. In particular, for all varying-coefficient functions, the estimates (dashed) are close to the true functions (solid) which mostly lie within the simultaneous (dotted) and pointwise (dashed-dotted) credible intervals. As expected, the performance of our method (e.g., smaller bias and narrower pointwise and simultaneous credible intervals) improved for the $n = 476$ regions case (Figure 1 in the main text).

Online Appendix B. Details on Data Analysis Results

In the data analysis, all Markov chains were verified to have good mixing and convergence properties and in Figure S3, we present the trace plots for the time-invariant

parameters $(\sigma^2_{u_1}, \sigma^2_{u_2}, \rho_u, \alpha_1, \alpha_2)$. We also examined the scale reduction factor, R, to monitor convergence, as suggested by Gelman and Rubin (1992), and confirmed that $R \approx 1$.

Online Appendix C. Model Fit Assessment

To assess our joint model fit, we utilized the deviance information criterion (DIC), a Bayesian measure that penalizes model complexity (Spiegelhalter et al., 2002; Gelman et al., 2014). DICs were computed for the following simpler models:

(M1) – includes time-varying regression coefficients and ignores the spatial dependency

$$m_{ij}(t) = g^{-1}\{\mathbf{X}_{ij}^{\mathrm{T}}\beta_y(t) + \mathbf{Z}_i^{\mathrm{T}}\gamma_y(t) + u_{1ij}\}$$
$$h_{ij}(t \mid \mathbf{X}_{ij}, \mathbf{Z}_i, u_{2ij}, v_{2i}) = h_0(t)\exp\{\mathbf{X}_{ij}^{\mathrm{T}}\beta_s(t) + \mathbf{Z}_i^{\mathrm{T}}\gamma_s(t) + u_{2ij}\}.$$

(M2) – incorporates the spatial dependency through an MCAR correlation structure and includes time-invariant regression coefficients, that is, ignores temporal patterns

$$m_{ij}(t) = g^{-1}\{\mathbf{X}_{ij}^{\mathrm{T}}\beta_y + \mathbf{Z}_i^{\mathrm{T}}\gamma_y + u_{1ij} + v_{1i}\}$$
$$h_{ij}(t \mid \mathbf{X}_{ij}, \mathbf{Z}_i, u_{2ij}, v_{2i}) = h_0(t)\exp\{\mathbf{X}_{ij}^{\mathrm{T}}\beta_s + \mathbf{Z}_i^{\mathrm{T}}\gamma_s + u_{2ij} + v_{2i}\}.$$

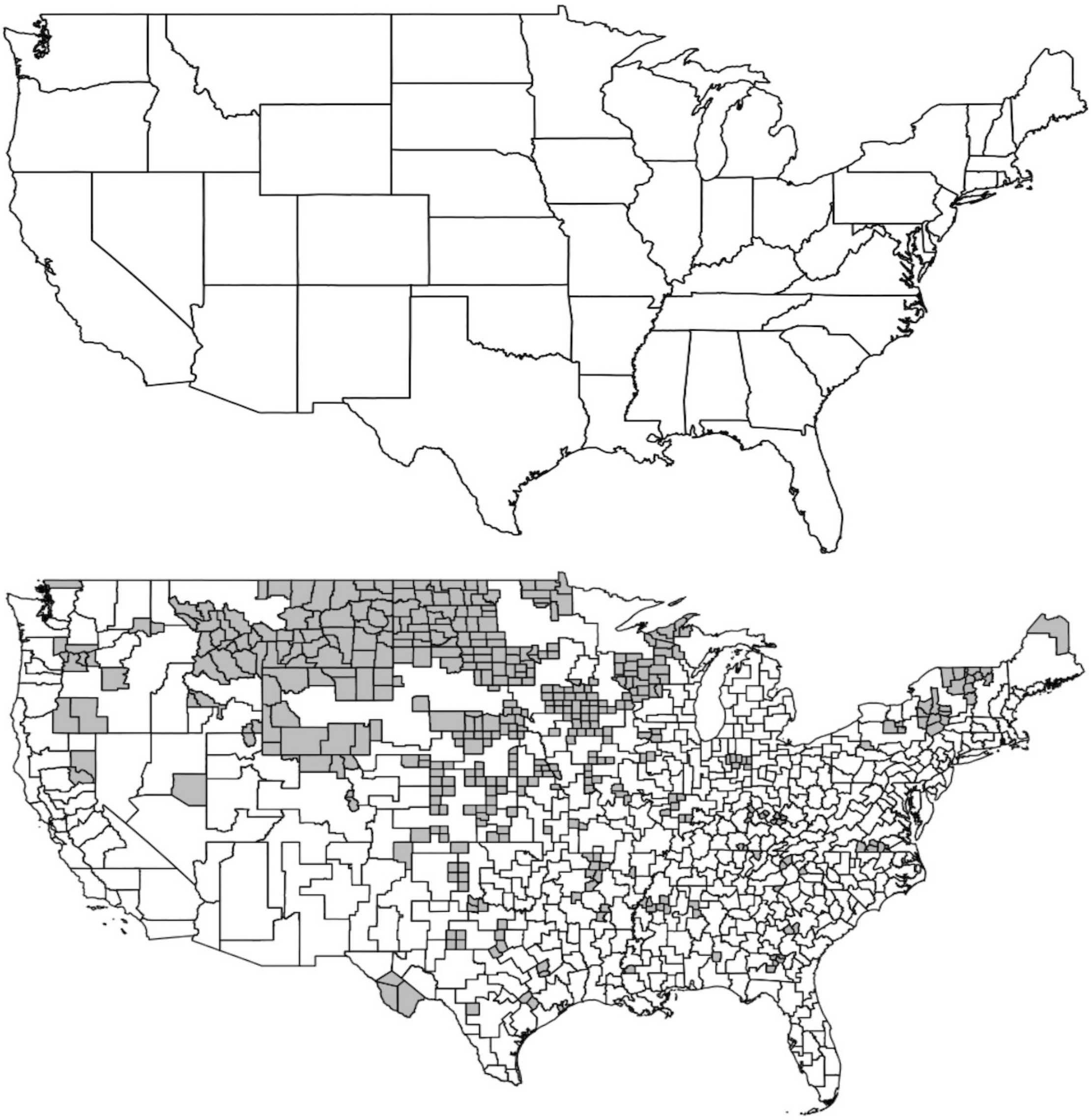

Fig. S1 Maps of $n = 49$ states (top) in the contiguous U.S. (including the District of Columbia) and $n = 476$ health services areas (HSAs) (bottom).

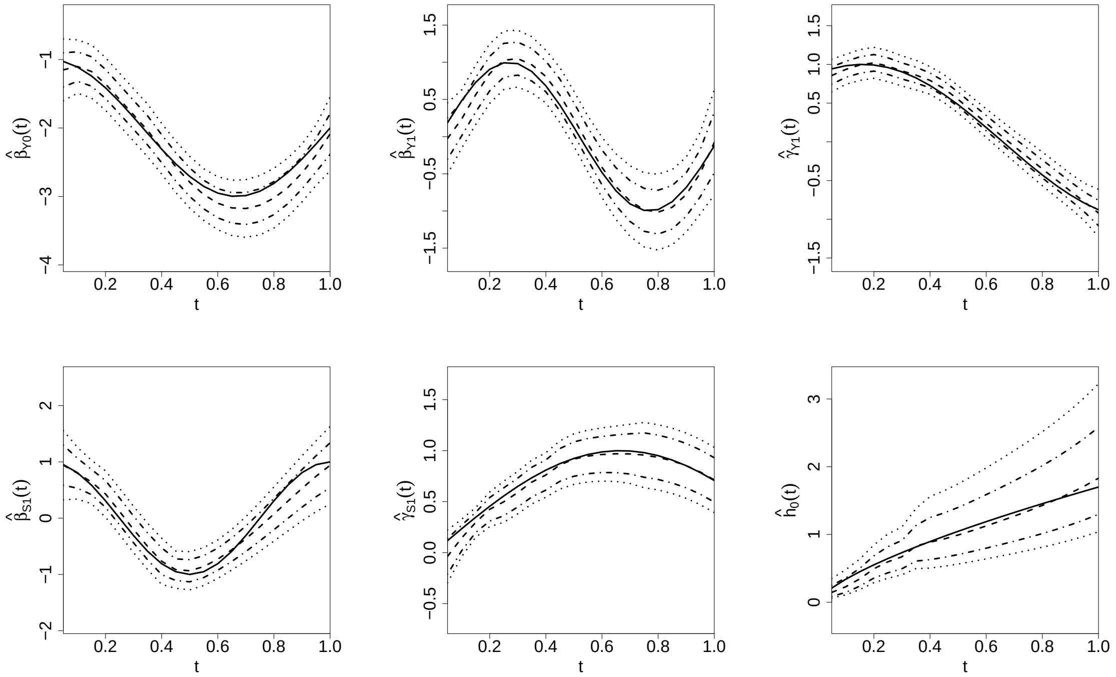

Fig. S2 Estimated time-varying coefficient functions (dashed) in the longitudinal submodel (top row) and survival submodel (bottom row) from the simulation runs with median RASE among 150 Monte Carlo runs for $n = 49$ regions overlaying the true functions (solid) along with 95% simultaneous (dotted) and pointwise (dashed-dotted) credible intervals.

(a) $\sigma^2_{u_1}$

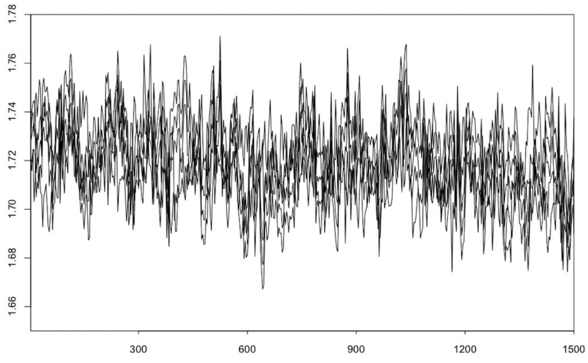

(b) $\sigma^2_{u_2}$

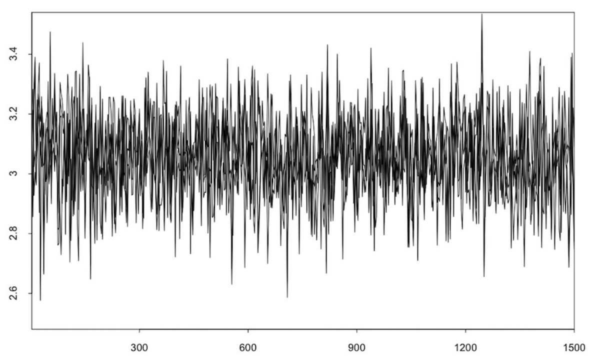

(c) ρ_u

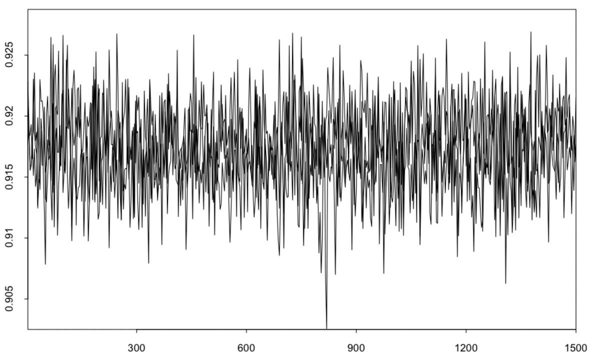

(d) α_1

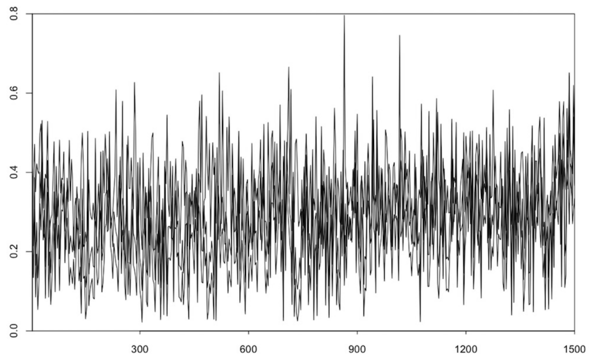

(e) α_2

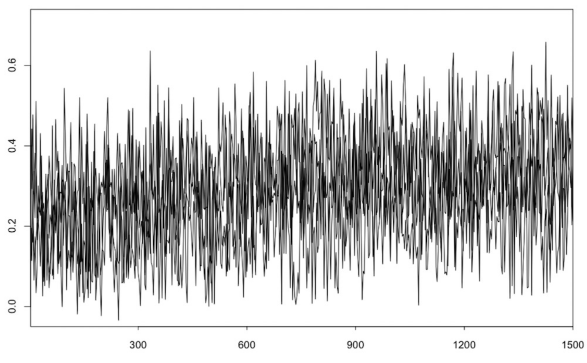

Fig. S3 MCMC trace plots for time-invariant parameters. Posterior samples were obtained by running three parallel chains with 6000 iterations per chain, where 1500 iterations were discarded as burn-in and the thinning was selected to keep 1500 posterior samples in each chain.

References

Gelman A, Rubin DB (1992) Inference from iterative simulation using multiple sequences. Statistical science 7(4):457–472

Gelman A, Carlin J, Stern H, Dunson D, Vehtari A, Rubin D (2014) Bayesian data analysis, vol. 2 crc press. Boca Raton, FL

Spiegelhalter DJ, Best NG, Carlin BP, Van Der Linde A (2002) Bayesian measures of model complexity and fit. Journal of the royal statistical society: Series b (statistical methodology) 64(4):583–639
